# Methods of Machine Learning-Based Chimeric Antigen Receptor Immunological Synapse Quality Quantification

**DOI:** 10.1007/978-1-0716-3135-5_32

**Published:** 2023

**Authors:** Julian Gan, Jong Hyun Cho, Ryan Lee, Alireza Naghizadeh, Ling Yue Poon, Ethan Wang, Zachary Hui, Dongfang Liu

**Keywords:** Chimeric antigen receptor (CAR), Immune synapse, Immunological synapse, IS, glass-supported lipid bilayer, SLB, Confocal microscopy, Artificial neural networks (ANN)

## Abstract

Chimeric Antigen Receptor (CAR)-mediated immunotherapy shows promising results for refractory blood cancers. Currently, six CAR-T drugs have been approved by U.S. Food and Drug Administration (FDA). Theoretically, CAR-T cells must form an effective immunological synapse (IS, an interface between effective cells and their target cells) with their susceptible tumor cells to eliminate tumor cells. Previous studies show that CAR IS quality can be used as a predictive functional biomarker for CAR-T immunotherapies. However, quantification of CAR-T IS quality is clinically challenging. Machine learning (ML)-based CAR-T IS quality quantification has been proposed previously.

Here, we show an easy-to-use, step-by-step approach to predicting the efficacy of CAR-modified cells using ML-based CAR IS quality quantification. This approach will guide the users on how to use ML-based CAR IS quality quantification in detail, which include: how to image CAR IS on the glass-supported planar lipid bilayer, how to define the CAR IS focal plane, how to segment the CAR IS images, and how to quantify the IS quality using ML-based algorithms.

This approach will significantly enhance the accuracy and proficiency of CAR IS prediction in research.

## 1 Introduction

Chimeric antigen receptor (CAR) immunological synapse (IS) is the interface between CAR-modified cells and their susceptible target cells [1, 2]. This interface includes several key steps of CAR-modified cell activation and cytotoxicity. Specifically, these steps include (1) the initiation of CAR-T/NK cell engaging with tumor cells, (2) activation of CAR signaling, (3) mobilization of cytotoxic machinery (e.g., lytic granules) into the IS area, and (4) degranulation and killing of tumor cells via an effective IS between CAR-T cells and tumor cells. The IS formation by CAR-T cells begins upon tumor antigen-specific interaction with CAR. This initial contact forms a cluster of tumor antigens, analogous to the central cluster of the T-cell receptors (TCR) at the synapse [3]. After accumulating the CAR-tumor antigen complexes in combination with other co-stimulatory molecules in the IS, these clusters can trigger the activation of CAR signaling, beginning with the phosphorylation of the intracellular downstream signaling molecules, such as CD3ζ.

Additionally, phosphorylation and micro-clustering of other signaling molecules of the TCR, such as ZAP70 and Lck, are important indicators of CAR-T cell activation [4, 5]. Following CAR activation, re-organization, and accumulation of the F-actin ring around the synapse stabilizes the IS and leads to the polarization of lytic granules, such as perforin and granzymes, to the synapse [6,7]. The functional CAR IS formation can lead to degranulation of the cytotoxic granules and efficient killing of tumor cells via a synapse. Thus, CAR IS’s effective formation and quality can be imaged and quantified [8,9].

**Fig. 1:**
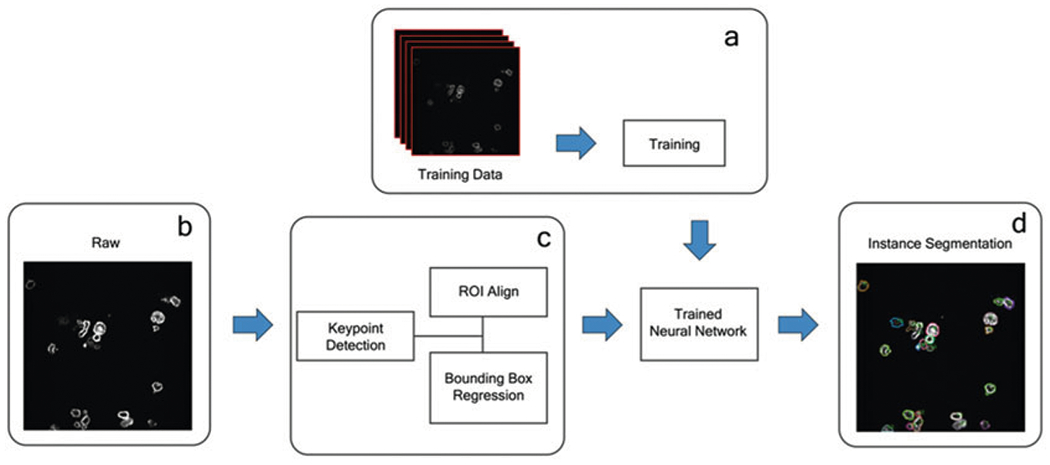
Illustrative Key Steps of CAR IS Image Processing. Using neural networks requires manually annotating training datasets by exposing the program to large amounts of training data. (**a**) Once trained, image processing can begin. Raw images (**b**) are processed through a series of machine learning algorithms (**c**) used to identify cell instances and later to quantify each cell. The final processed image (**d**) shows the computer’s identification of individual cells

**Fig. 2:**
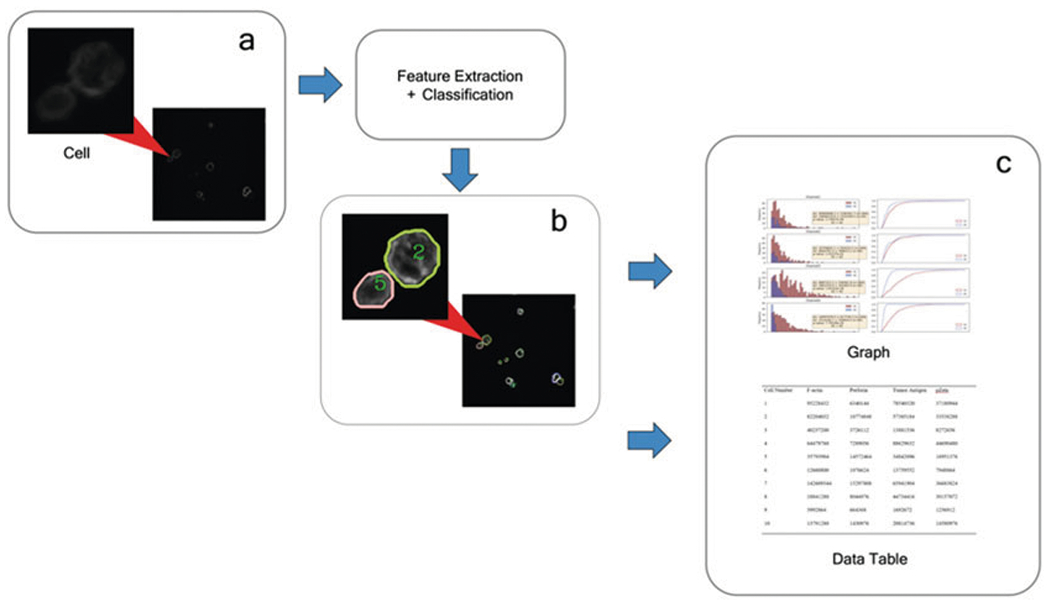
Illustrative model of CAR IS quantification. (**a**) The machine processes raw images (**b**) to identify cell instances. (**c**) From this, quantification data is automatically transferred to an Excel sheet and graphed (**c**)

**Fig. 3:**
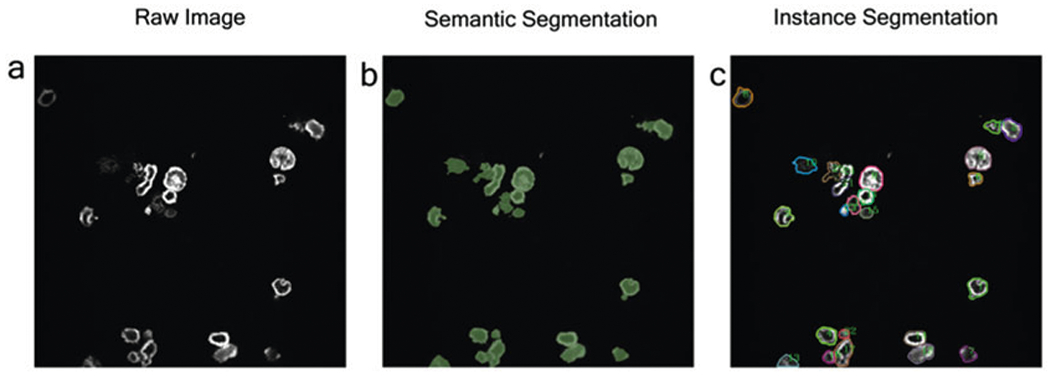
Comparison between semantic and instance segmentation. (**a**) Raw image input to be processed. (**b**) Semantic segmentation identifies objects within an image by differentiating each pixel as a cell or non-cell. (**c**) Instance segmentation differentiates cell instances, classifying each cell as its instance of an object

Through a new development inspired by machine learning (ML), our team applies the practice of instance segmentation [8, 10] to high-resolution CAR IS images (Fig. 1), highlighting the CAR-modified cells and quantifying their fluorescence intensities. Using neural networks that perform pattern recognition algorithms, object detection, and cross-validation, our program automates the process of CAR IS quantification (Fig. 2). Keypoint detection is used to find the top-left, top-right, bottom-left, bottom-right, and center of each cell individually. Bounding boxes generated using Keypoint detection have proven to be more accurate than previous methods [11]. When a bounding box generation is complete, non-maximum suppression is applied to prevent multiple detections of the same object [12]. There are two methodologies of identifying cells-semantic segmentation and instance segmentation (Fig. 3). Semantic segmentation methods identify multiple objects within the same category as one object. On the other hand, instance segmentation distinguishes individual cells as unique objects.

Introducing instance segmentation to imaging CARs has proven more effective and efficient than other ML methods in immunotherapy [8]. With this approach, our team improves on conventional ML methods with more accurate results on any density of cells. By utilizing pre-built deep learning modules such as artificial neural networks (ANNs) and their various configurations [13], our program is easy to install and simple to use.

Applying these principles yields a high-throughput system, leading to a faster evaluation of CAR IS quality. Our program also reduces labor costs and marginal error. We aim to create a user-friendly method of quantifying CAR IS data while improving cost and efficacy.

## 2 Materials

The ML-based CAR IS quantification software has hardware and software requirements as described below.

### 2.1 Recommended Hardware Configuration

Compute Unified Device Architecture (CUDA)-capable NVIDIA graphics card is recommended (*see* Note 1). Since CUDA is backward compatible, older NVIDIA graphics card series may be used without issue.

### 2.2 Software Installation

1. Download CAR IS quantification software from Google Drive’s file sharing platform: https://drive.google.com/drive/folders/1NJRHLD_dUPYQgwVngwhvhZm1-xt4mK3F?usp=sharing
2. You can obtain the software by downloading “Package1” and “checkpoint.pth.tar”
3. Download and install the latest version of Python 3 from: https://www.python.org/downloads/.
4. Open the command terminal and enter the installation command for each library (Table 1) (*see* Note 2).
5. Install CUDA toolkit from: https://developer.nvidia.com/cuda-downloads (*see* Note 3).
6. Install the cuDNN compatible with your CUDA toolkit from: https://developer.nvidia.com/cudnn.

## 3 Methods

### 3.1 Immunofluorescence Imaging of CAR IS

1. As previously described, preparing the glass-supported planar lipid bilayer with CAR-NK cells is important for IS data acquisition [8]. To acquire images, turn on all the necessary hardware and software modules for Nikon A1R confocal microscope with a motorized stage. Choose 60’ 1.4 NA oil objective and select the desired fluorescent channels and their laser settings.
2. To find the CAR IS focal plane, identify the highest intensity peak of the tumor antigen channel. Upon CAR interaction, the tumor antigen clustering on the lipid bilayer can best capture the IS focal plane.
3. Set the Z-stack to 0.25 μm per slice for 5 slices relative to the focal plane position (*see* Note 4).
4. Choose 10–20 regions of interest (ROIs) within the lipid bilayer to analyze.
5. Start acquiring images of the CAR IS with the motorized stage (*see* Note 5).
6. Export the images to 16-bit TIFF format to quantify the CAR IS quality.

### 3.2 Automated CAR IS Quantification Using Machine Learning

**Fig. 4:**
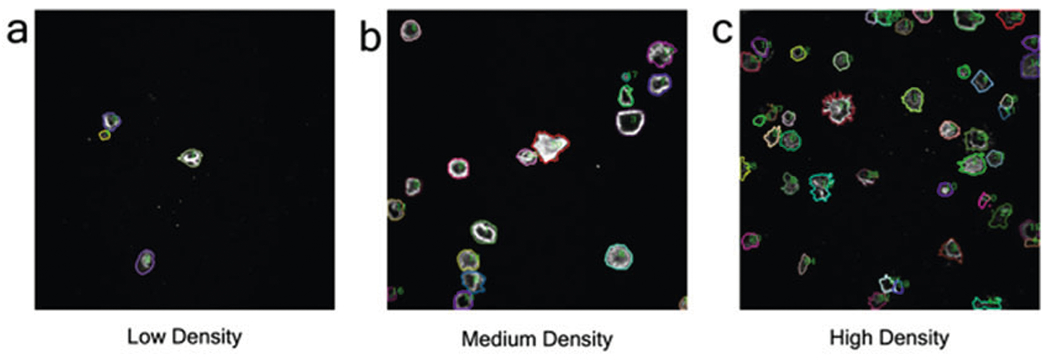
Visual representation of instance segmentation on CAR IS. (**a**–**c**) Each image represents a different level of cell cluster density

**Fig. 5:**
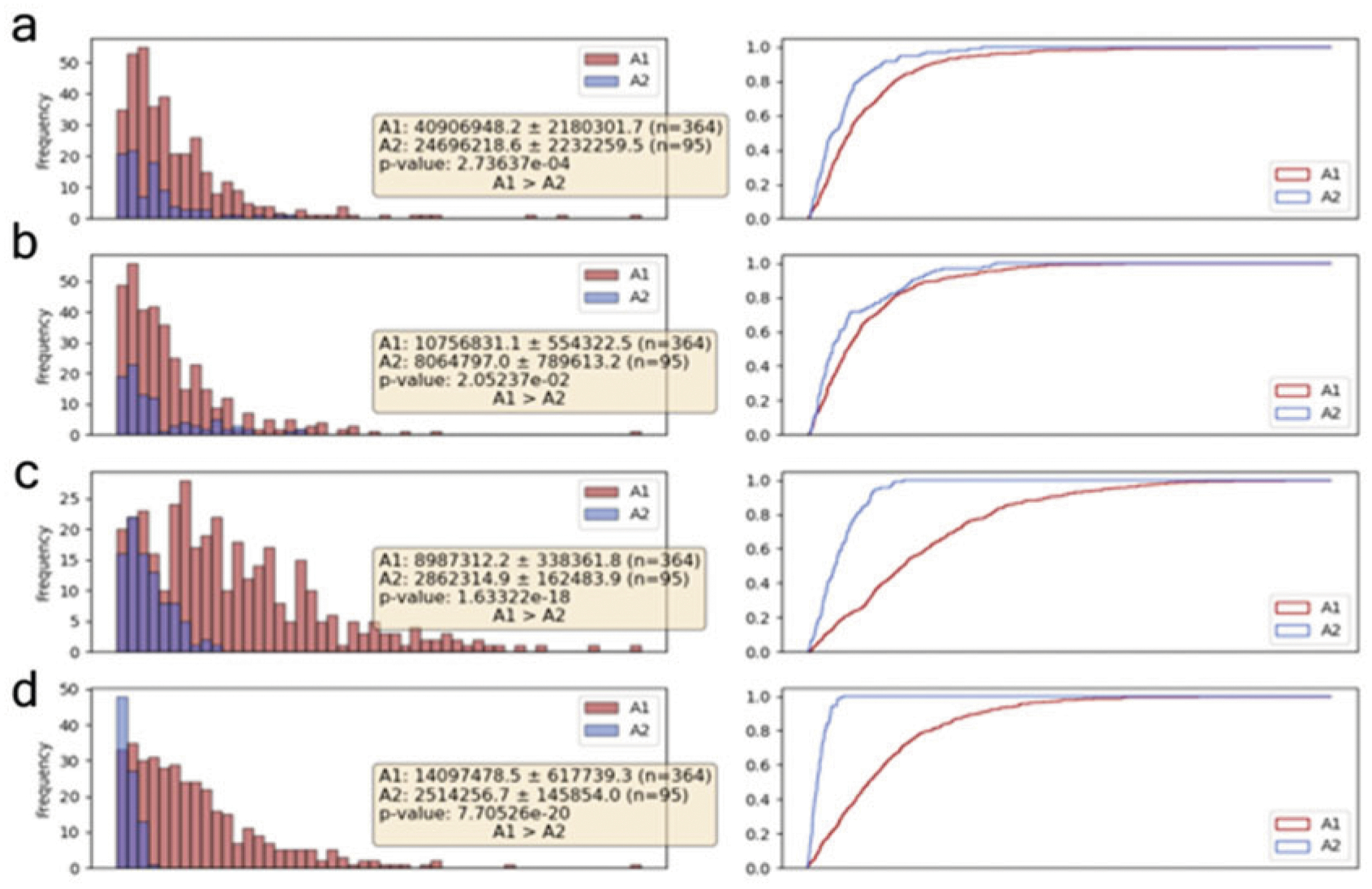
Quantification of the CAR IS quality using our ML-based algorithm. Each row represents specifically designated channels and shows the total intensity distribution. In this case, (**a**) Channel 1 is F-actin, (**b**) Channel 2 is perforin, (**c**) Channel 3 is IgG_1_ Kappa antigen, and (**d**) Channel 4 is phospho-CD3ζ. A1 represents Kappa-CAR NK92, and A2 represents untransduced (UTD) NK92. (**a**–**d**) The bar graphs on the left show the number of cells (frequency) against total fluorescence intensity. The line graphs on the right show the cumulative probability against total fluorescence intensity. The figures also show the mean, variance, and the number of cells detected. The p-value is calculated with an unpaired t-test

The following steps cater to Linux operating systems. A user-friendly interface is developing when writing this paper (*see* Note 6):

1. From the package provided in Subheading 2.2 **item 1**, Open the *run* file using a text editor such as Notepad (*see* Note 7).
2. Line 2 of the *run* file, after “cd,” update the following file location to the folder containing *run* (*see* Note 8).
3. Line 4 determines where processed image results will be saved. Update the folder location within the single quotes to the preferred folder path.
4. Line 5, within the single quotes, update the folder location to the folder containing unprocessed image data.
5. On line 6, enter the order of the images from the image data folder. After each channel name is a space followed by a number representing the order of the channel. “0” represents the first channel in order, “1” represents the second channel and so on.
6. On line 7, write the names of the folders containing the images that need to be compared. For example, “—compares ‘folder1’ ‘folder2’ \,” with “folder1” and “folder2” each being a folder containing ordered image data.
7. Click on “Save” to save the changes made to the *run* file.
8. Open a terminal (*see* Note 9) and type “cd” followed by the folder location of the package, and press “enter.”
9. Type and enter “./run” to run the program. Results (Fig. 4) can be saved to the directory specified in **step 3**. Quantification results (Fig. 5) involve the total fluorescence intensity (Table 2), mean fluorescence intensity (MFI), and the synapse area.

### 3.3 Alternative Method: Manual Evaluation of CAR IS Using ImageJ

1. Open *.nd2* file using ImageJ.
2. Separate each channel by choosing “Images,” select “Color,” and “Split Channels.”
3. Under “Images,” select “Stacks” and then “Plot Z-axis Profile” to identify the brightest focal plane.
4. Choose the three brightest focal planes and combine them into a single image by navigating under “Image,” selecting “Stacks,” and then “Z Project.”
5. To quantify the MFI at the synapse, go to “Analyze,” select “Tools,” and “ROI Manager.”
6. On the DIC slide, circle the cells individually, selecting “Show All” and “Labels.” Then transfer these labels to other channel images.
7. Select “Measure” and set measurements, selecting “Area,” “Mean,” and “Integrated Density.”
8. To calculate the background intensity, repeat **steps 5–7**, creating 5 ROIs (4 corner regions and a center region) for each channel background. Subtract the background mean value from the MFIs of the synapse.

## Figures and Tables

**Table 1:** List of commands for the installation of required Python libraries. Each row provides the library name, the required installation command, and a short description of its function. To install one of the following libraries, open a terminal on Linux or the command prompt on Windows, copy and paste the installation command, and press “enter”

Library	Installation command	Summary
NumPy	pip3 install NumPy	Collection of machine-learning mathematical functions
PyTorch	pip3 install torch torchvision torchaudio	Deep learning and computer vision framework allows for both CPU and GPU processing
Pillow	pip3 install pillow	Support for image processing and manipulation
OpenCV	pip3 install OpenCV-python	Collection of additional computer vision functions
SciPy	pip3 install scipy	Collection of optimization and scientific computation algorithms
Natsort	pip3 install natsort	Number sorting tool
Nd2Reader	pip3 install nd2reader	Tool for reading Nikon NIS elements images
Xlwt	pip3 install xlwt	Writes and formats data for Microsoft excel
Xlrd	pip3 install xlrd	Reads data from Microsoft excel files
Tifffile	pip3 install tifffile	Reads and writes image data in multiple formats
Scikit-image	pip3 install scikit-image	Collection of image processing algorithms
Pandas	pip3 install pandas	Data analysis and manipulation software
Matplotlib	pip3 install matplotlib	Data visualization and graphing tool

**Table 2:** Sample data illustration. The machine learning program will generate an Excel file containing the total fluorescence intensity of each cell in an image

Cell Number	F-actin	Perforin	Tumor antigen	pZeta
1	95,228,432	6,340,144	78,540,320	37,180,944
2	82,204,032	10,774,848	57,385,184	33,538,288
3	40,237,200	3,726,112	13,881,536	8,272,656
4	64,479,760	7,289,056	88,629,632	44,690,480
5	35,793,984	14,572,464	34,842,096	16,951,376
6	12,660,800	1,076,624	13,759,552	7,948,864
7	142,609,344	15,297,808	65,941,904	36,683,824
8	18,841,280	8,044,976	44,734,416	30,157,072
9	3,992,864	664,368	1,692,672	1,256,912
10	13,791,280	1,430,976	20,814,736	14,580,976

## 4 Notes

1. If using an NVIDIA graphics card is impossible, you may also use the CPU version of CUDA installable from the PyTorch official website. Select the “CPU” variant of the installation package.

2. The use of a Python virtual environment is not necessary but recommended.

3. Check the compatibility of the CUDA toolkit to your NVIDIA driver version. An NVIDIA driver update may be required. For more information, see: https://docs.nvidia.com/deploy/cuda-compatibility/index.html.

4. To take the 3D image of the synapse, we take two slices up and down from the focal plane, each Z-stack step at 0.25 μm for a total of 1 μm. The number of Z-stacks can be increased or decreased accordingly.

5. For faster acquisition of the images on the Nikon A1R microscope, you can choose the resonant scan option with Denoise.ai for noise removal.

6. Our development team works on a graphical user interface (GUI) while writing this paper. A GUI will provide a simpler way to access and use the machine learning program.

7. Text editors such as Visual Studio Code are recommended as they provide greater functionality and are easy to use.

8. The run folder is also simply the unzipped package we provide in Subheading 2.2 **item 1**. To view this folder’s location, right-click the folder and select “properties.”

9. On Linux operating systems, open the menu and search “terminal.” On Windows, search “cmd” to open the Command Prompt.
